# Molecular and Physiological Evaluation of Bread Wheat (*Triticum aestivum* L.) Genotypes for Stay Green under Drought Stress

**DOI:** 10.3390/genes13122261

**Published:** 2022-11-30

**Authors:** Ahmad Zada, Ahmad Ali, Dalal Nasser Binjawhar, Usama K. Abdel-Hameed, Azhar Hussain Shah, Shahid Maqsood Gill, Irtiza Hussain, Zaigham Abbas, Zahid Ullah, Hassan Sher, Iftikhar Ali

**Affiliations:** 1 College of Bioscience and Biotechnology, Yangzhou University, Yangzhou 225009, China; 2 Department of Botany, Hazara University, Mansehra 21120, Pakistan; 3 Center for Plant Sciences and Biodiversity, University of Swat, Charbagh 19120, Pakistan; 4 Department of Chemistry, College of Science, Princess Nourah Bint Abdulrahman University, Riyadh 11671, Saudi Arabia; 5 Biology Department, College of Science, Taibah University, Al-Madinah Al-Munawarah 42353, Saudi Arabia; 6 Botany Department, Faculty of Science, Ain Shams University, Cairo 11566, Egypt; 7 Department of Biotechnology and Genetic Engineering, Hazara University, Mansehra 21120, Pakistan; 8 Land Resources Research Institute, National Agricultural Research Centre, Islamabad 45500, Pakistan; 9 Department of Biochemistry, PMAS Arid Agriculture University, Rawalpindi 46000, Pakistan; 10 Department of Biological Sciences, International Islamic University, Islamabad 44000, Pakistan; 11 Department of Genetics and Development, Irving Medical Center, Columbia University, New York, NY 10032, USA

**Keywords:** drought stress, stay green, *LHCI*, *LHCII*, gene expression

## Abstract

Water availability is considered as the main limiting factor of wheat growth illuminating the need of cultivars best adapted to drought situations for better wheat production and yield. Among these, the stay-green trait is thought to be related to the ability of wheat plants to maintain photosynthesis and CO_2_ assimilation, and a detailed molecular understanding of this trait may help in the selection of high-yielding, drought-tolerant wheats. The current study, therefore, evaluated the physiological responses of the selected wheat genotypes under pot-induced water stress conditions through different field capacities. The study also focused on exploring the molecular mechanisms involved in drought tolerance conferred due to the stay-green trait by studying the expression pattern of the selected PSI-associated light-harvesting complex I (LHC1) and PSII-associated LHCII gene families related to pigment-binding proteins. The results revealed that the studied traits, including relative water content, membrane stability index and chlorophyll, were variably and negatively affected, while the proline content was positively enhanced in the studied wheats under water stress treatments. Molecular diagnosis of the selected wheat genotypes using the expression profile of 06 genes, viz. *TaLhca1*, *TaLhca2*, *TaLhca3*, *TaLhcb1*, *TaLhcb4* and *TaLhcb6* that encodes for the LHCI and LHCII proteins, indicated variable responses to different levels of drought stress. The results obtained showed the relation between the genotypes and the severity of the drought stress condition. Among the studied genotypes, Chirya-1 and SD-28 performed well with a higher level of gene expression under drought stress conditions and may be used in genetic crosses to enrich the genetic background of common wheat against drought stress.

## 1. Introduction

Wheat (*Triticum aestivum* L.) is a major cereal crop and an integral part of daily diet in different geographic regions of the world [1]. In the past three decades, unpredictable climatic conditions have resulted in stagnated wheat production. Wheat yield and quality are highly impacted by multiple factors including cultivar types and abiotic stresses such as high temperature, drought stress etc., [2,3,4]. About 37% of the semiarid area belongs to developing countries, where available moisture is primarily used for wheat cultivation [5]. Drought or water stress can be defined as the shortage of adequate moisture needed for normal growth of a plant growth and complete life cycle [6]. It is a major environmental stress, which can affect growth of cereal crops and decrease the production and performance of the plant [7,8,9]. It is considered a big hazard for sustainable agriculture and is becoming a worldwide trouble [10,11,12,13]. A significant rise in drought stress conditions has enforced us to develop climate-resilient, high-yielding genotypes; hence, there is a need to acquire a better understanding of the responses to drought by exploiting the key physiological and molecular traits [14,15,16]. Further, it has been estimated that wheat yield can be increased by 25% through the development of abiotic and biotic stress-tolerant cultivars [17,18,19,20,21]. 

The term “stay green” is mostly used for mutants with decreased chlorophyll catabolism [22]. The capacity of certain genotypes in several annual crop species including wheat to maintain green leaves during the grain-filling period (the “stay-green” phenotype, SG) is an intriguing crop feature, especially under drought stress conditions. It is considered an important trait that allows plants to retain their leaves in active photosynthetic state when subjected to stress conditions [2,11,23,24]. Direct evidence for the contribution of this trait to high-yield potential is lacking. Therefore, a greater understanding of this trait is needed to explore possible selection of SG because it is expected to have a significant role in the productivity of wheat, particularly under harsh environments [25,26].

Two main types of chlorophyll are present in higher plants, Chlorophyll a and b, where the former is a part of all chlorophyll–protein complexes, whereas the later is confined only in PSI-associated light-harvesting complex I (LHCI) and PSII-associated LHCII. Light-harvesting complexes I and II are present in thylakoid membranes and their function is energy production and transfer. LHCII is mostly present in grana and, due to intermolecular forces, its main function is the formation and maintenance of grana stacks [27,28,29]. The *Lhca* and *Lhcb* gene families encoded the apoproteins of light-harvesting complex I and II, respectively. The *Lhca1*–*Lhca4* genes formed the protein of LHCI, which are associated with PSI. The *Lhcb1*, *Lhcb2*, and *Lhcb3* genes code the polypeptides of trimeric LHCII. The *Lhcb4*, *Lhcb5*, and *Lhcb6* proteins (also known as CP29, CP26, and CP24,) are proposed to be monomeric proteins, which are found one set per PSII unit [30]. The *Lhca* and *Lhcb* genes expression and LHCI and LHCII stability are very important to keep the photosynthetic process at high level [31,32,33]. The present study aimed to explore the interaction of drought stress with stay-green expression through the physiological responses in wheat genotypes. Molecular characterization through changes from transcription and translation of genes encoding major functional proteins to PSI and PSII under progressive water stress was also focused.

## 2. Methods and Materials

### 2.1. Greenhouse Experiment

The experimental plant materials comprised two conventional wheats, i.e., Opata and Chirya-1, and two synthetic derivatives i.e., SD-28 and SD-32 having *Aegilops squarrosa* in its pedigree (ESM 1). Opata is a relatively non-stay-green, drought-susceptible wheat variety; SD-28 and SD-32 are moderately stay-green drought-tolerant wheat genotypes, while Chirya-1 is a stay-green, drought-tolerant wheat cultivar. The selection was based on a 2-year, detailed characterization of a focus collection of 325 diverse bread wheats for water stress effects on stay green and chlorophyll fluorescence, with a focus on its yield characteristics for two years (data unpublished). A pot experiment was conducted under greenhouse conditions with temperatures from 10 °C to 15 °C and 13 to 14 h light duration, to assess the effect of progressive drought stress, including control (100%), moderate stress (80%) and severe stress (60% field capacity i.e., FC). Field soil, sand and peat compost were mixed in equal proportion and filled in 36 pots at the rate of 1835 g in each pot. Nine pots were used for each wheat genotype replicated three times with three different field capacities (i.e., three pots per treatment arranged in randomized complete block design). The pots were tapped on the floor enough to bring the soil mixture to a uniform height to achieve the soil bulk density (SBD) and the corresponding total soil porosity (TSP).

The height of the soil mixture in each pot required to achieve the desired SBD was calculated according to Jiang et al. [34] by following formula.

$$\mathrm{Height}\;(\mathrm{cm})=\frac{\mathrm{VSM}\;({\mathrm{cm}}^{3})}{\pi \times \frac{{\mathrm{Diameter}\;\mathrm{of}\;\mathrm{pot}\;(\mathrm{cm})}^{2}}{2}}$$

where VSM (volume of soil mixture) was calculated by the following formula:

$${\mathrm{VSM}\;(\mathrm{cm}}^{3})=\frac{\mathrm{Mass}\;\mathrm{of}\;\mathrm{soil}\;\mathrm{mixture}\;\mathrm{taken}\;\mathrm{in}\;\mathrm{each}\;\mathrm{pot}\;(g)}{{\mathrm{Desired}\;\mathrm{SBD}\;(g\;\mathrm{cm}}^{-3})}$$

The pots were irrigated to saturate all the pore space of the soil mixture and kept aside for drying. The volume of water (VW) required for saturating the soil mixture in the pots was calculated by the formula

$${\mathrm{VM}\;(\mathrm{cm}}^{3}{)=\mathrm{TSP}\;(\mathrm{cm}}^{3}{\;\mathrm{cm}}^{-3})\;\times {\;\mathrm{VSM}\;(\mathrm{cm}}^{3})$$

where

$${\mathrm{TSP}\;(\mathrm{cm}}^{3}{\;\mathrm{cm}}^{-3})=\frac{{\mathrm{SBD}\;(g\;\mathrm{cm}}^{-3})}{{\mathrm{SPD}\;(g\;\mathrm{cm}}^{-3})}$$

The volumetric soil water content (VSWC) was monitored daily from each pot with a Time Domain Reflectometer (TRIME –FM-IMKO Micromodultechnik GmbH, Ettlingen, Germany), and the seeds were sown in the pots when the VSWC was at FC (assuming FC as 50% of the saturation and taking saturation = TSP).

$${\mathrm{VSWC}\;\mathrm{at}\;100\%\;\mathrm{FC}\;(\mathrm{cm}}^{3}{\;\mathrm{cm}}^{-3})=\frac{{\mathrm{TSP}\;(\mathrm{cm}}^{3}{\;\mathrm{ncm}}^{-3}\times \;50)}{100}$$

Similarly, the VSWC at other drought levels was calculated with their respective formulae according to Zhang et al. [35].

$${\mathrm{VSWC}\;\mathrm{at}\;80\%\;\mathrm{FC}\;(\mathrm{cm}}^{3}{\;\mathrm{cm}}^{-3})=\frac{{\mathrm{TSP}\;(\mathrm{cm}}^{3}{\;\mathrm{ncm}}^{-3}\times \;40)}{100}$$

$${\mathrm{VSWC}\;\mathrm{at}\;60\%\;\mathrm{FC}\;(\mathrm{cm}}^{3}{\;\mathrm{cm}}^{-3})=\frac{{\mathrm{TSP}\;(\mathrm{cm}}^{3}{\;\mathrm{ncm}}^{-3}\times \;30)}{100}$$

After germination, three seedlings were raised per pot, for which the VSWC was monitored and maintained daily at 100% FC for three weeks. The VW required for maintaining each pot at FC was calculated by the formula:

VW (cm^3^) = (VSWC at 100% FC (cm^3^ cm^−3^) − VSWC from TDR (cm^3^ cm^−3^)) × VSM (cm^3^)

When wheat seedlings were 21 d old, the irrigation supply was cut off from the pots to which the drought treatment was applied, and the VSWC was monitored daily until the respective drought level was achieved.

VW (cm^3^) = (VSWC at 80% FC (cm^3^ cm^−3^) − VSWC from TDR (cm^3^ cm^−3^)) × VSM (cm^3^)

VW (cm^3^) = (VSWC at 60% FC (cm^3^ cm^−3^) − VSWC from TDR (cm^3^ cm^−3^)) × VSM (cm^3^)

The drought stress was maintained for 30 d till visible wilting in plants grown at 60% FC, after which the uppermost, fully expanded three-leave sample per plant from all pots was collected for further physiological and molecular investigation.

### 2.2. Physiological Characterization

The studied physiological traits included the leaf relative water content [36], the leaf chlorophyll content [37], the leaf membrane stability index [38] and the proline content determination [39]. The stay-green trait was measured following the method of Xu et al. [40] and Silva et al. [41] by scoring on a 1–5 scale, based on the percentage area of the normal-sized leaves that had senesced and died early.

### 2.3. Molecular Characterization of Wheat Genotypes

The genes related to the pigment-binding proteins were selected to examine the different expressions in the four genotypes of wheat that were grown under progressive drought stress including control (100%), moderate stress (80%) and severe stress (60% field capacity. A total of six genes were studied and the specific primers for genes encoding LHCI and LHCII were designed based on the published expressed sequence tags. These included:

1. ACCGCCAGCTCTTCCACCCT (*Tubulin-*F); TCACTGGGGCATAGGAGGAA ((*Tubulin-*R);
2. TCAGCGACCTCACCGTCA (*TaLhcb6*-F); CCCCAAAGAAGTCACGGACA (*TaLhcb6-*R);
3. AAAGGCCGAGGAGGACAA (*TaLhcb4*-F); CCACCGACCACTTAAGAGG (*TaLhcb4*-R);
4. GGAGAACACACAATACACC (*TaLhcb1*-F); CCCATTATGTGTGCAGTTC (*TaLhcb1*-R);
5. CCTCACCAGCCTCAAGTTCC (*TaLhca3-F*); CGCACGCTCACGTTTCC (*TaLhca3-* R);
6. CCCCAACCGCAAGAACC (*TaLhca2*-F); CCGACGAAGGCGAGCAT (*TaLhca2*-R);
7. CAACCTGCCGACCATCCTG (*TaLhca1-*F) and CAGCCGCCCGTTCTTGAT (*TaLhca1-*R).

#### 2.3.1. Total RNA Extraction and cDNA Synthesis

Leaf samples were collected from each replication and immediately transferred to liquid nitrogen. The total RNA of the four wheat varieties grown under progressive drought stress including control (100%), moderate stress (80%) and severe stress (60% field capacity) was extracted in duplicate (Gene JET Plant RNA Purification Mini kit, Thermo Fisher Scientific, Waltham, MA, USA). The cDNA was synthesized using oligo dT primers (Revertaid reverse transcription kit, Thermo Fisher Scientific, Waltham, MA, USA). Briefly, 11 uL template RNA was mixed with 1.0 µL of Oligo (dt) 18 primers (#S0132) and incubated for 5 min at 65 °C. Then, 4.0 µL of RT buffer, 1 µL Ribolock Rnase inhibitor and 1 µL of reverse transcriptase (Revert Aid) and 2 µL of dNTPs mix (#R0181) were added to the total volume of 20 µL. The mixture was centrifuged and incubated at 42 °C for 80 min. The reaction was terminated by heating at 70 °C for 5 min, and the synthesized cDNA was stored at −20–42 °C for RT–PCR analysis. The whole process of RNA extraction and cDNA synthesis was accomplished according to the extraction and cDNA synthesis discussed in Kianersi et al. [42].

#### 2.3.2. Real-Time PCR and Data Analysis

The cDNA was amplified by real-time PCR using SYBR green dye (Thermo Fisher Scientific, Waltham, MA, USA). The specific primer sets for genes encoding LHCI (*TaLhca1*, *TaLhca2*, and *TaLhca3*) and LHCII (*TaLhcb1*, *TaLhcb4*, and *TaLhcb6*) pigment-binding proteins were designed based on the published expressed sequence tags. Amplification of the *T. aestivum tubulin* gene exhibiting constitutive expression was used as a reference/housekeeping gene control. Each target gene in both stressed and control plants was examined against the housekeeping gene. The amplification was carried out with a thermal profile: initial denaturation at 95 °C for 10 min followed by 35 cycles of denaturation at 95 °C for 20 s, annealing at 58 °C for 40 s and extension at 72 °C for 30 s. The data obtained from thermal cycler were analyzed by 2^−∆∆**Ct**^ method [43,44]. The amplified products obtained from qRT–PCR were separated using a 1% agarose gel to confirm the size of the desired products.

### 2.4. Statistical Analysis

Using completely randomized design (CRD), data obtained were subjected to descriptive statistics, analysis of variance and correlation test. The analysis of treatments was performed by using STATISTIX 10 (Analytical Software, Tallahassee FL, USA) and means was compared by using Fisher’s least significant difference (*p* < 0.01 and 0.05).

## 3. Results and Discussion

### 3.1. Relative Water Content (RWC)

The RWC in all the studied wheats was highly different in both control and drought stress treatments. Under control conditions, the mean RWC was 94.6% while under drought stress conditions, the mean values of RWC were 73.6% and 81.9% in plants grown at 60% and 80% FC, respectively showing a relative decline of 22.1% and 13.4% in RWC as compared to control condition (Table 1). The ANOVA revealed high differences in RWC among treatments, while the studied wheats and its interaction with the treatment yielded no significant differences (Table 2). The LSD indicated differential and promising behavior among the studied wheats in response to imposed drought stress, particularly Chirya-1 exhibited the least decrease in relative water content (19.9%) at 80% FC (Table 3). 

### 3.2. Membrane Stability Index (MSI)

The descriptive statistics of the studied attributed under water stress treatment is given in Table 1. Regarding the membrane stability index, an increasing trend was observed under moderate water stress (8.9%) which slightly decreased by 2.0% under 60% FC, in comparison to the control treatment. Further, the mean MSI in the control treatment was 55.2%, while it was 60.6 and 54.1% under the stress condition (80 and 60% FC, respectively). The results clearly indicated that the membrane stability was highly affected by drought stress. The analysis of variance also revealed a highly different MSI among the studied wheats and drought treatment (Table 2). Fischer’s LSD indicated no significant differences in the MSI under control treatment; however, under drought conditions of 80% and 60% FC, high MSI of 62.7% and 55.9% were recorded for SD-28 wheat genotype, respectively (Table 3).

### 3.3. Proline Contents

Descriptive statistics revealed that the mean proline content under 100% FC was 0.83 µmol/g, while it was 1.43 and 2.51% under the moderate and severe water stress treatment, respectively. Further, relatively increased PRCs of 41.9 and 66.9% were recorded under the moderate and severe stress treatment, respectively, when compared to plants grown in the control environment (Table 1). The ANOVA regarding proline content also yielded high differences among the studied wheats, water treatments and the interaction between them (Table 2). The LSD regarding the PRC also showed differential behavior in the studied wheats and water stress treatments. In comparison to the control, the relative increase in PRC of 61.6, 56.1, 72.3 and 73.35 in SD-28, SD-32, Opata and Chirya-1, respectively, was observed under the severe water stress treatment. Similarly, at 80% FC, the maximum PRC 1.67 µmol/g was recorded for stay-green Chirya-1 followed by SD-32 (Table 3). The results clearly demonstrated that water stress led to an enhanced, improved proline content and that the different wheats have different responses to various drought levels.

### 3.4. Chlorophyll Contents

Descriptive statistics also showed that chlorophyll content was affected by drought stress treatment. The observed mean chlorophyll a (Chla) was 1.19 mg/g under the control condition, whereas it was 1.04 and 0.54 mg/g under the moderate and severe stress treatments. Further, under moderate drought stress, the maximum Chla content of 1.09 mg/g was recorded for SD-32, while under severe drought stress (60% FC), it was 0.57 mg/g in SD 28 followed by the stay-green Chirya-1 wheat. Likewise, the mean chlorophyll b (Chlb) was 1.11 mg/g in plants grown under the control environment, while 0.56 and 0.93 mg/g were recorded under the progressive water stresses, respectively (Table 1). Under the control condition and moderate stress, the maximum Chlb (1.25 and 1.16 mg/g) was recorded for the stay-green Chirya-1 wheat, while under severe stress it was 0.58 mg/g in SD 28 followed by the stay-green Chirya-1 wheat cultivar. Similarly, mean total chlorophyll (TChl) under the control environment was recorded as 2.29 mg/g, while it was 1.97 and 1.10 mg/g under moderate and severe drought stress, respectively. The analysis of variance also yielded high differences among the studied wheats, water treatments and the interaction between them (Table 2). Fisher’s LSD depicted highly differential behaviors among the studied wheats in response to the different water treatments. In comparison to the control, the least relative decrease was observed in the SD-28 and stay-green Chirya-1 (11.6 and 12.2 %, respectively) in the moderate stress treatment. By contrast, under the severe drought stress treatment, the least relative decrease of 47.2 and 49.3% was observed in the SF-28 and SD-32 wheats, respectively. The results indicated that the stay-green Chirya-1, SD-28 and SD-32 wheats performed well under drought stress condition.

### 3.5. Expression Levels of Genes Involved in LHCI and LHCII in Response to Drought Stress

The six genes that are responsible for the coding pigment-binding proteins were studied to find the different transcriptional responses under the control conditions of 100% FC and the different level of drought stress condition i.e., 80% FC and 60% FC, respectively, in the two synthetic-derived wheat genotypes (SD-28 and SD-32) and the two check cultivars (Chirya-1 and Opata). 

**Figure 1:**
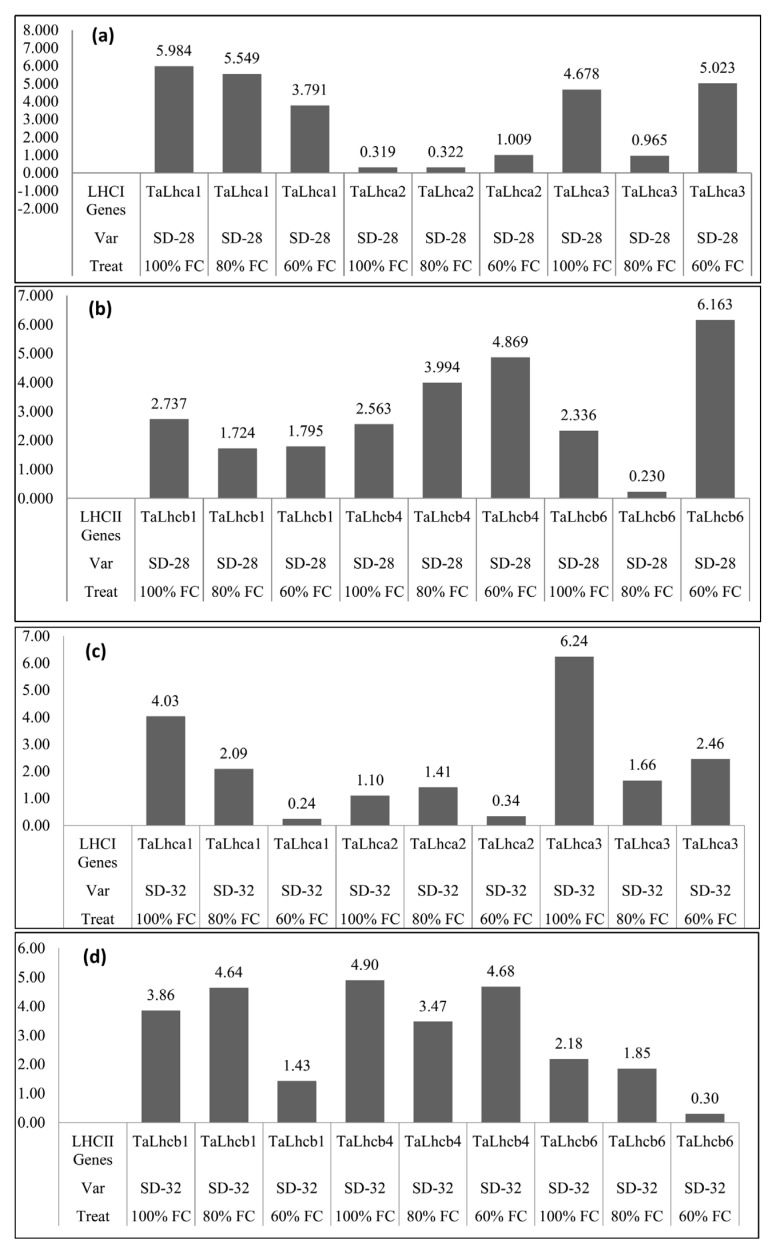
Expression of (**a**) *LHCI* genes under different drought levels in SD−28, (**b**) *LHCII* genes under different drought levels in SD−28, (**c**) *LHCI* genes under different drought levels in SD−32, (**d**) *LHCII* genes under different drought levels in SD−32.

All genes showed a differential expression level in SD-28 genotype. The *TaLhca1* gene showed downregulation in both 60% and 80% FC but highly suppressed in 60% FC condition, while the *TaLhca2* gene showed upregulation in 60% FC but its expression in 80% FC and control plants was approximately same. *TaLhca3* is downregulated in both stress conditions but exhibits a higher suppression level in 80% FC. The *TaLhcb1* gene equally downregulated under both conditions. The *TaLhcb4* showed upregulation in both 80% FC and 60% FC. The expression of *TaLhcb6* was also upregulated under both stress conditions but the expression was higher in 60% FC. (Figure 1a,b).

In the SD-32 genotype genes, *TaLhca1*, *TaLhca3* and *TaLhcb4* showed downregulation in both 80% and 60% FC. The *TaLhca2* gene was equally upregulated in both stress conditions. The gene *TaLhcb1* was upregulated in 80% FC, but its downregulation was observed in 60% FC. Similarly, the gene *TaLhcb6* exhibits upregulation in 80% FC but its downregulation was revealed in a variety at 60% FC (Figure 1c,d).

**Figure 2:**
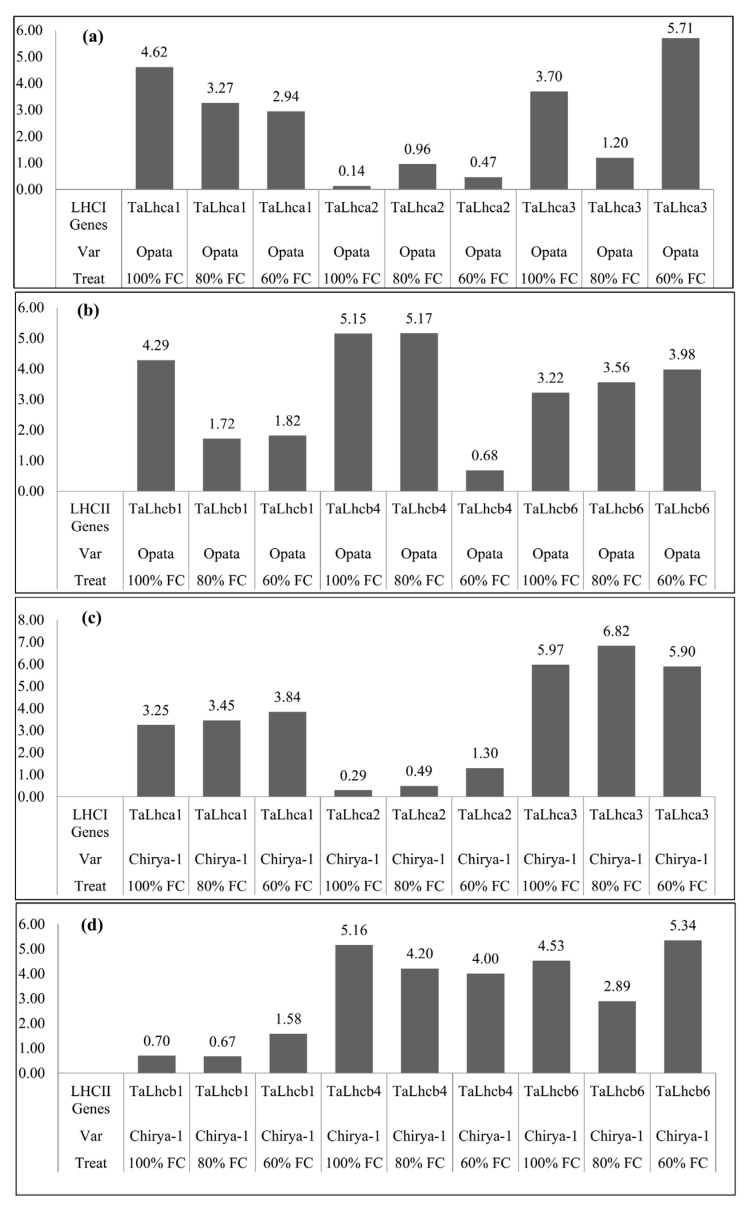
Expression of (**a**) *LHCI* genes under different drought levels in Opata, (**b**) *LHCII* genes under different drought levels in Opata, (**c**) *LHCI* genes under different drought levels in Chirya−1, (**d**) *LHCII* genes under different drought levels in Chirya−1.

The expression level of genes that encode proteins for the pigment-binding molecule LHCI showed highly significant results in the SG Chirya-1 genotype. All the genes that involved in LHCI, namely *TaLhca1*, *TaLhca2* and *TaLhca3*, show a higher level of expression and their expressions were not affected by drought stress condition. Similarly, the genes that involve in LHCII, namely *TaLhcb1*, *TaLhcb4* and *TaLhcb6*, also show similar results such as those involved in LHCI. The expression levels of these genes were not significantly affected by drought stress conditions, which are evident from the result that the SG Chirya-1 genotype is drought resistant as compared to the Opata and synthetic-derived genotypes. The studied physiological traits also indicated that Chirya-1 performed well under drought stress condition. In the Opata, for both stress conditions only the *TaLhca2* gene expression was enhanced, whereas the expression of all other genes was suppressed except for *Talhcb4* in 80% FC, where its expression remains the same as in the control. In the Chirya-1 genotype, all the genes were upregulated in both stress conditions except for *TaLhca1* and *TaLhcb1* in 80% FC, where these genes showed downregulation (Figure 2a–d).

### 3.6. Discussion

The four selected wheats exhibited high differences for the studied traits under the different drought treatments. Leaf RWC indicates the ability of plants to keep their water status adequate to sustain water stress. In our results, the RWC declined during water stress in all the studied wheats under the different drought stress levels. Additionally, it has been shown that the RWC is negatively correlated with the membrane stability index, and the larger the water loss from leaves manifested by smaller the RWC, the greater will be the cell membrane damage [45,46]. Our results are consistent with the findings of Almeselmani et al. [47] and Kocheva et al. [48] that drought stress sufficiently decreases the water status, osmotic potential and nutrient uptake of plants, which finally results in a low leaf turgor pressure and reduced metabolic activities. Under a water-deficit environment, the membrane stability is known as an important factor to tolerate drought stress, and hence this factor is considered vital during selection of plants to cope with unfavorable conditions [49]. In our results, different drought levels highly affected the membrane stability, leading to water loss from plant tissues, which in turn seriously impaired both membrane structure and function. Our current results are supported by the previous observation of Narayanan [50] and Sayar et al. [51]. Khalvandi et al. [52] have also reported the correlation of electrolyte leakage with drought tolerance.

Increased accumulation of proline is indicative of plant adaptive responses to water stress environments and its increased accumulation is considered beneficial as an osmoticum and desiccation protectant [53,54,55,56]. We observed an increased proline content with an increase in the severity of drought stress. These results are in agreement with the previous studies by Parida et al. [57] and Kaur and Asthir [58].

Changes in photosynthetic pigments may led to an alteration in photosynthetic efficiency, including the capability to harvest light, hence to observe these pigments is essential to determine the plant photosynthetic effectiveness. In the current research, a differential decreasing trend was observed in the chlorophyll content; however, the decrease was mild, which may be due to the increased osmoprotectant and different tolerance levels of the studied wheats. Reports of such slightly altered or mild decreased chlorophyll contents have been reported by Wasaya et al. [59] and Khayatnezhad and Gholamin [60]. Several other researchers have also reported harm to the leaf chlorophyll content in response to drought stress [11,61,62,63,64].

In this study, the effect of drought stress on the expression of genes, which encode LHCI and LHCII related proteins, was observed in four varieties of wheat. The RT–PCR data suggested the differential expression of these genes, but the expression of each gene was affected by the drought stress. Tian et al. [30] observed no significant differences in the expression pattern of *Talhca1*, *Talhca3* and *Talhcb1* genes between the wild type and Tasg1a, however *Talhcb4*, *Talhcb6* genes were upregulated and the *Talhca2* gene was downregulated in the Tasg1 stay-green mutant. In our study, we found that all the studied genes showing significant differences between the wild type and stress-induced plants confirming that all these genes might be involved in drought stress-related functions. It can be conferred that the introgression of the favorable alleles for stress tolerance has taken place from the diploid wild species *Aegilops taushcii*, as previously reported [53,65,66].

The stay-green mutants are characterized by an increased stability of chloroplast membranes and chlorophyll–protein complexes [32,67]. Current research mainly focused on the effects of the stay-green trait regarding wheat responses under water stress. The SG trait was noted to have a direct effect on the proline content, membrane stability index, chlorophyll and relative water content, which exhibited high differences among the studied wheat genotypes for the SG ratings. In a previous work, it was observed that the expression level of the genes in the SG genotype was comparatively greater under drought condition [68]. The results are in agreement with the previous findings of Ullah et al. [1], Petrov et al. [55], Hussain et al. [64] and Brestic et al. [69,70]. The results also supported the findings of Zhao et al. [71] regarding gene expression levels under drought stress condition during photosynthesis. The retention of LHCII is thought to play an important role in the formation and maintenance of grana stacks [31]. In this study, the expression levels of the two genes encoding the LHCII proteins, *TaLhcb4* and *TaLhcb6*, were found to be higher in tasg1 compared with the WT, especially at the 48 h time point under drought stress. The results obtained in previous experiments indicated the involvement of abiotic stress responses in connections with further levels of regulation at post-transcriptional and post-translational levels. The overall results regarding the level of genes expression are in general agreement with Oksman-Caldentey and Saito [72] and Reinders and Sickmann [73]. Previously, Tian et al. [68] reported comparatively enhanced chlorophyll b content (52.0 and 72.5%) in the SG genotype under well-watered and drought stress treatment, suggesting its probable contribution to the maintenance of grana stacks. Our findings suggested that further exploration of these genes could lead to the development of phenotypes that resist the drought stress up to significant levels.

## 4. Conclusions

It is concluded from current research that progressive drought stress had substantial effects on the physiological responses of the studied wheats. Photosynthetic pigments and relative water contents exhibited a declining trend with an increase in the severity of drought stress. It was observed that the overexpression of genes, viz. *TaLhca1*, *TaLhca2*, *TaLhca3*, *TaLhcb1*, *TaLhcb4* and *TaLhcb6* that encode for the LHCI and LHCII proteins, was directly proportional to the drought stress. Among the studied genotypes, the wheat SG Chirya-1 and synthetic-derived (SD-28) performed well with a better physiological performance accompanied with a higher level of gene expression under drought stress condition. Hence, these were considered drought-tolerant genotypes with the potential to enrich the genetic background of locally adapted wheat lines against drought stress.

## Figures and Tables

**Table 1:** Descriptive statistics of the studied physiological attributes under different field capacities.

Variables	Mean	SD	Variance	SE Mean	C. V.	Minimum	Maximum
Membrane Stability Index (%)							
100% FC	55.23	3.55	12.62	1.03	6.43	49.37	59.38
80% FC	60.69	1.98	3.91	0.57	3.26	57.21	62.83
60% FC	54.11	5.01	25.12	1.45	9.26	43.94	62.26
Proline Content (µmol/g)							
100% FC	0.83	0.09	7.95	0.03	10.77	0.64	0.93
80% FC	1.43	0.24	0.06	0.07	16.87	1.01	1.68
60% FC	2.51	0.47	0.22	0.14	18.81	1.71	3.19
Relative Water Content (%)							
100% FC	94.58	2.51	6.28	0.72	2.65	91.25	98.32
80% FC	81.89	2.37	5.69	0.69	2.91	77.63	86.61
60% FC	73.66	2.87	8.24	0.83	3.88	70.34	79.34
Chlorophyll a (mg/g)							
100% FC	1.19	0.04	1.52	0.01	3.28	1.12	1.26
80% FC	1.04	0.04	1.91	0.01	4.19	0.99	1.14
60% FC	0.54	0.04	1.38	0.01	6.85	0.47	0.59
Chlorophyll b (mg/g)							
100% FC	1.11	0.17	0.03	0.05	15.72	0.85	1.34
80% FC	0.93	0.16	0.02	0.04	16.79	0.72	1.18
60% FC	0.56	0.04	1.24	0.01	6.29	0.52	0.62
Total Chlorophyll (mg/g)							
100% FC	2.29	0.18	0.03	0.052	7.84	2.04	2.57
80% FC	1.97	0.14	0.02	0.041	0.04	1.79	2.20
60% FC	1.10	0.05	2.85	0.015	4.85	0.99	1.20

**Table 2:** Analysis of variance (ANOVA) results for the studied physiological traits under control and water stress condition.

SOV	DF	MSI	Proline	RWC	Chla	Chlb	Total Chl
Treatments	2	148.9 ***	8.692 ***	1333 ***	1.371 ***	0.938 ***	4.563 ***
Wheats	3	21.82 ^NS^	0.244 ***	7.35 ^NS^	0.004 **	0.120 ***	0.106 ***
Treatments * Wheats	6	20.07 ^NS^	0.323 ***	7.38 ^NS^	0.004 ***	0.033 ***	0.031 ***
Error	22	11.980	0.022	7.380	0.001	0.002	0.031

where, ** and *** depict statistical significance at 0.01 and 0.001 probability level, respectively; * is used for interaction; SOV, source of variation; ^NS^, non-significant; MSI, membrane stability index; RWC, relative water content; Chla, chlorophyll a; Chlb, chlorophyll b; TChl, total chlorophyll.

**Table 3:** Least significant differences of the studied physiological traits under control and water stress condition. Different letters indicate significant statistical differences according to Fisher’s LSD test at 0.05 probability level.

Treatment	Wheat	MSI	Proline	RWC	Chla	Chlb	TChl
100% FC	SD-28	58.176 ^ab^	0.872 ^h^	93.701 ^a^	1.200 ^ab^	0.914 ^cd^	2.115 ^b^
100% FC	SD-32	50.679 ^c^	0.864 ^h^	96.477 ^a^	1.168 ^bc^	0.985 ^c^	2.153 ^b^
100% FC	Opata	53.637 ^bc^	0.857 ^h^	93.010 ^a^	1.144 ^c^	1.285 ^a^	2.431 ^a^
100% FC	Chirya-1	58.415 ^ab^	0.719 ^h^	95.131 ^a^	1.230 ^a^	1.247 ^a^	2.477 ^a^
80% FC	SD-28	62.685 ^a^	1.148 ^g^	82.303 ^b^	1.017 ^e^	0.850 ^de^	1.869 ^c^
80% FC	SD-32	62.047 ^a^	1.556 ^ef^	80.193 ^bc^	1.099 ^d^	0.777 ^e^	1.876 ^c^
80% FC	Opata	59.268 ^ab^	1.358 ^fg^	82.175 ^b^	1.034 ^e^	0.917 ^cd^	1.952 ^c^
80% FC	Chirya-1	58.764 ^ab^	1.667 ^e^	82.876 ^b^	1.014 ^e^	1.159 ^b^	2.173 ^b^
60% FC	SD-28	55.966 ^bc^	2.272 ^c^	73.396 ^de^	0.576 ^f^	0.562 ^f^	1.115 ^d^
60% FC	SD-32	53.740 ^bc^	1.971 ^d^	71.46 ^e^	0.547 ^f^	0.542 ^f^	1.090 ^d^
60% FC	Opata	54.707 ^bc^	3.091 ^a^	73.659 ^de^	0.492 ^g^	0.562 ^f^	1.055 ^d^
60% FC	Chirya-1	52.009 ^c^	2.698 ^b^	76.111 ^cd^	0.550 ^f^	0.592 ^f^	1.143 ^d^

For trait code, see Table 2.

## Data Availability

Not applicable.
